# Safety, accuracy, empathy, information quality, and readability of publicly accessible LLM-based chatbots for traumatic brain injury and concussion questions: a cross-sectional comparative study

**DOI:** 10.3389/fpubh.2026.1926980

**Published:** 2026-09-01

**Authors:** Xin Zuo, Huan Zuo, Min Zhang, Weihong Zheng

**Affiliations:** 1 Department of Acupuncture and Moxibustion, Heilongjiang University of Traditional Chinese Medicine, Harbin, China; 2 Department of Neurology, Xiamen Humanity Hospital, Fujian Medical University, Xiamen, Fujian, China

**Keywords:** chatbots, concussion, large language models, patient education, traumatic brain injury

## Abstract

**Background:**

Large language model (LLM)-based chatbots are increasingly used by the public to obtain health information, but their performance in answering questions related to traumatic brain injury (TBI) and concussion remains unclear. This study evaluated five publicly accessible LLM-based chatbots across safety, accuracy, empathy, information quality, transparency, and readability.

**Methods:**

Sixty-five English-language, public-facing questions about TBI and concussion were submitted to ChatGPT, Gemini, Copilot, DeepSeek, and Doubao, generating 325 responses. Five blinded independent raters assessed the generated responses using guideline-informed criteria and established tools, including DISCERN, EQIP, JAMA benchmark criteria, the Global Quality Score, and readability indices.

**Results:**

Inter-rater agreement was high. The proportions of responses rated as safe ranged from 89.2 to 95.4%, and all models achieved a median accuracy score of 4.00. However, 27 responses were classified as potentially harmful, mainly because of under-triage, overly reassuring advice regarding imaging findings, premature return-to-activity or driving guidance, and insufficient pediatric caution. Accuracy differences were statistically significant but small, whereas empathy, information quality, transparency, and readability showed clearer model-level variation. DeepSeek produced the easiest-to-read responses.

**Conclusion:**

LLM-based chatbots generated responses that were generally rated as safe and informative by expert evaluators, but potentially harmful advice and readability problems remained. These findings characterize expert-rated response performance and do not establish patient comprehension, educational effectiveness, or clinical benefit. LLM-based chatbots may have potential as supplementary sources of patient-facing health information, but they should not replace professional medical evaluation.

## 1 Introduction

Traumatic brain injury (TBI) is a major global public health problem and one of the leading causes of death, disability, emergency department visits, and long-term neurological sequelae worldwide (1–3). The clinical spectrum of TBI is broad, ranging from mild concussion to severe intracranial injury requiring urgent neurosurgical care (4, 5). Although most TBIs are classified as mild, even mild TBI or concussion may be associated with persistent headache, dizziness, cognitive difficulties, sleep disturbance, mood symptoms, delayed return to school or work, and reduced quality of life (6–8). Because early warning signs may be subtle and symptoms can evolve over time, patients and caregivers frequently seek practical guidance on whether emergency evaluation is needed, whether computed tomography (CT) or magnetic resonance imaging (MRI) is appropriate, how to observe symptoms at home, and when it is safe to resume driving, work, school, exercise, or sports (9–13).

High-quality patient education is particularly important in TBI because inappropriate reassurance or delayed care may lead to missed intracranial bleeding, under-recognition of neurological deterioration, unsafe medication use, premature return to high-risk activity, or inadequate pediatric observation (9–14). Clinical guidelines and decision rules emphasize that TBI-related advice should distinguish low-risk symptoms from emergency red flags, consider age-specific and anticoagulant-related risks, avoid rigid self-triage thresholds, and provide clear instructions for follow-up and return precautions (9–12, 15, 16). At the same time, patient-facing information must be understandable and actionable. Low health literacy and overly complex medical information can reduce comprehension, impair shared decision-making, and limit adherence to recommended care (17–19). Therefore, evaluating TBI health information requires attention not only to factual accuracy, but also to safety, empathy, reliability, information quality, and readability.

Large language model (LLM)-based chatbots are increasingly used by the public to obtain health information. These systems can generate fluent, conversational, and individualized responses to medical questions, and previous studies have shown their potential in medical education, clinical knowledge tasks, and patient-facing communication (20–22). However, their apparent fluency may conceal important limitations, including hallucinated or incomplete information, inconsistent risk communication, lack of source transparency, and variable alignment with clinical guidelines (21–24). In urgent or safety-sensitive conditions such as TBI, these limitations are clinically relevant because users may rely on chatbot-generated answers when deciding whether to seek emergency care, observe symptoms at home, use medications, drive, exercise, or allow a child to return to school or sports.

Existing evaluations of online health information and AI-generated medical responses commonly use multidimensional tools, including DISCERN for reliability of treatment information, EQIP for patient information quality, JAMA benchmark criteria for transparency and accountability, global quality ratings, empathy assessment, and readability indices (25–28). Such multidimensional evaluation is well suited to TBI-related public consultation because a response may be factually adequate but insufficiently safe, empathetic but medically incomplete, or comprehensive but too difficult for patients to understand. However, evidence remains limited regarding how widely available LLM-based chatbots perform when answering common public questions about TBI and concussion across these complementary domains.

Therefore, this cross-sectional comparative study evaluated five widely used LLM-based chatbots based on their responses to public-facing health questions related to TBI and concussion. We assessed their performance across safety, accuracy, empathy, reliability, information quality, and readability using guideline-informed reference standards and established evaluation instruments. By identifying strengths and limitations across models, this study aimed to inform the responsible evaluation and potential use of LLM-based chatbots in patient-facing TBI health communication, rather than to establish their educational effectiveness or clinical benefit.

## 2 Methods

### 2.1 Study design

This was a cross-sectional comparative evaluation of publicly accessible LLM -based chatbots in response to public health consultation questions related to TBI and concussion. The study was designed to assess model performance across safety, accuracy, empathy, reliability, information quality, and readability. The reporting structure followed the CHART checklist for health-related AI chatbot evaluation where applicable (29). The completed checklist is provided in Supplementary material 1. Because this study evaluated chatbot-generated responses to standardized public-facing questions, the unit of analysis was the model-question response. No patient or public participants were involved in question development, prompt testing, response evaluation, or interpretation of the findings. Because the study used publicly formulated questions and did not involve human participants, patient records, identifiable personal information, or clinical interventions, formal ethics approval was not required according to local policy.

### 2.2 Development of the question set

The public consultation question set was developed by two investigators, Huan Zuo (HZ) and Xin Zuo (XZ). Candidate questions were collected from common public-facing TBI and concussion concerns, including emergency red flags, symptom interpretation, pediatric head injury, imaging decisions, home observation, medication use, daily activities, treatment and follow-up, return to school, work, sports, long-term sequelae, and prevention. The question sources included consultation themes identified from clinicians’ routine outpatient and emergency practice, public patient education materials, online health consultation topics, search-engine suggestions, and frequently asked questions from reputable health information resources.

To improve reproducibility, candidate questions were collected between 2026/6/16 and 2026/6/22 from the following sources: common outpatient and emergency consultation themes independently recalled and summarized by investigators with clinical experience in TBI and concussion care; patient education pages from authoritative medical or public health organizations; public online health consultation topics; frequently asked question sections of reputable health information websites; and search-engine autocomplete or “people also ask” suggestions. The clinical consultation themes reflected questions repeatedly encountered during routine practice and were incorporated into the initial question pool after discussion and consensus between the two investigators. No formal medical records, institutional databases, identifiable patient information, or individual patient cases were reviewed or extracted for this purpose. Search terms included “traumatic brain injury,” “TBI,” “concussion,” “head injury,” “mild traumatic brain injury,” “child head injury,” “brain injury CT scan,” “concussion return to sports,” “concussion driving,” and “post-concussion symptoms.”

**Figure 1:**
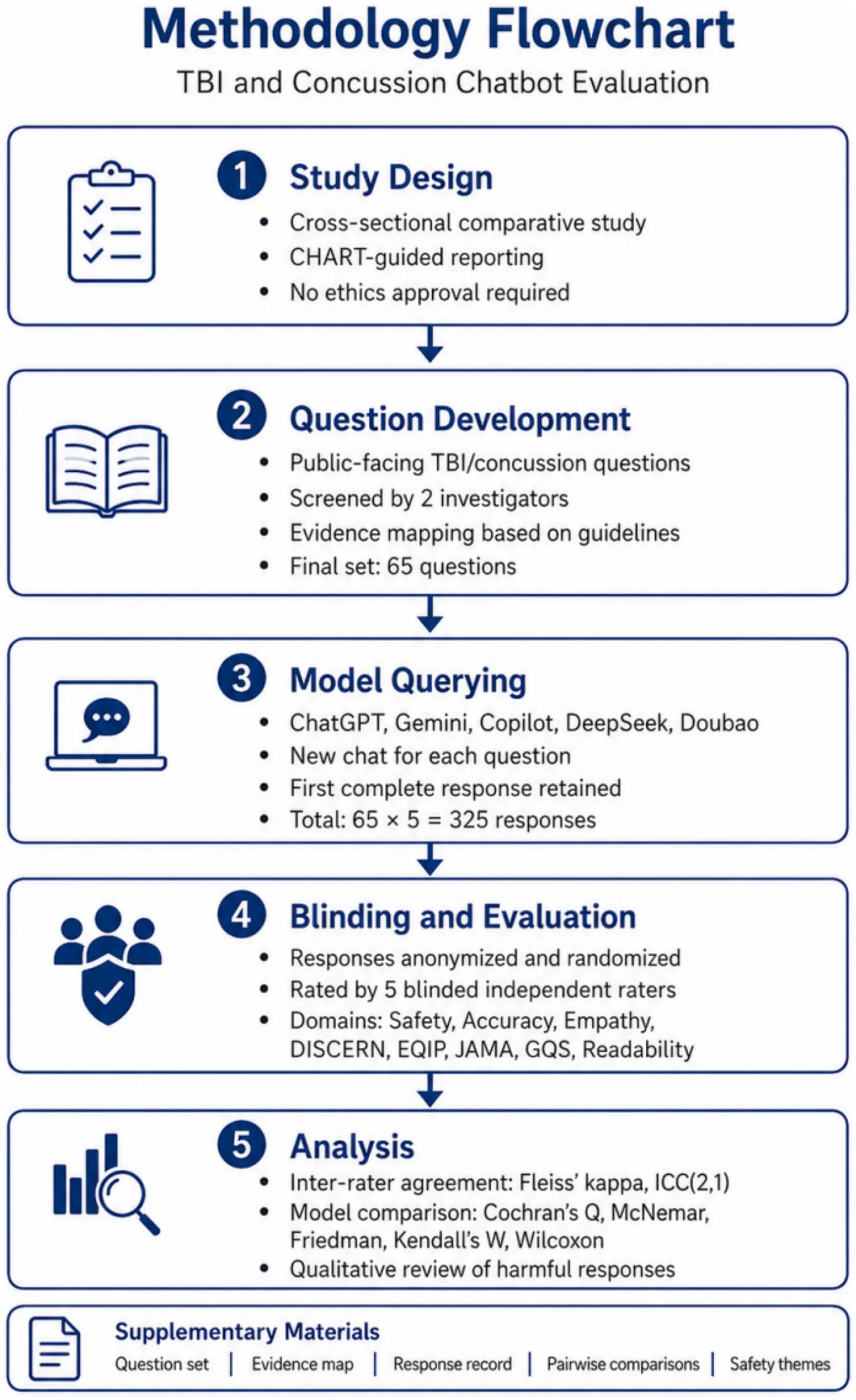
Methodological workflow of the cross-sectional comparative evaluation of LLM-based chatbots for TBI and concussion-related public questions.

A total of 119 candidate questions were initially identified and screened for eligibility. After screening for relevance, redundancy, clarity, generalizability, and suitability for standardized evaluation, 54 questions were excluded, leaving 65 questions in the final question set. Questions were excluded if they were unrelated to TBI or concussion, clinician-oriented, commercially oriented, dependent on images or unavailable clinical data, excessively individualized, or otherwise unsuitable for guideline- or consensus-based evaluation. The detailed screening process, including the number of questions excluded for each reason, is provided in Supplementary material 2 and summarized in Figure 1.

HZ and XZ independently screened the candidate questions. Questions were eligible if they were patient- or caregiver-facing, directly related to TBI or concussion, answerable using guideline- or consensus-based evidence, and relevant to public health consultation. Questions were excluded if they were duplicate or near-duplicate items, unrelated to TBI, written primarily for clinicians, commercially oriented, dependent on images or unavailable clinical data, or so individualized that a general educational answer would be inappropriate. Disagreements were resolved through discussion, and unresolved items were reviewed by a senior clinician, MZ or WZ, when necessary. The final included question set is provided in Supplementary material 2.

### 2.3 Evidence-based answer mapping

For each included question, two investigators (HZ and XZ) independently mapped the expected key answer elements to current guidelines, consensus statements, decision rules, and authoritative reviews on TBI and concussion (5, 6, 9–13). The mapping emphasized clinically important information such as emergency warning signs, indications for urgent evaluation or imaging, pediatric risk features, safe home observation, medication precautions, graded return to school, work, and sports, and follow-up after discharge. Differences between the two investigators were resolved by consensus, with senior clinical review when needed. The final question-answer evidence mapping table is provided in Supplementary material 3.

The evidence-mapping table included the standardized question, key expected answer elements, safety-critical points, common misconceptions or potentially unsafe omissions, and supporting guideline or review sources. This table served as the reference standard for rating accuracy and safety.

### 2.4 Model selection and prompting procedure

Five widely used publicly accessible LLM-based chatbots were selected because they are commonly available to general users and are likely to be used for health-related information seeking. The evaluated models included ChatGPT, Gemini, Copilot, DeepSeek, and Doubao. The specific model names, access modes, interface settings, dates of access, and geographic location of testing are summarized in Table 1. Exact backend model versions or release identifiers were recorded when publicly disclosed; when these details were unavailable, the model or mode label displayed in the user interface was reported together with the access date and route.

**Table 1:** LLM-based chatbots evaluated in this study.

Chatbot	Provider	Publicly displayed model/mode	Mode/setting	Access route	Query date	Query location	Interface language
ChatGPT	OpenAI	GPT-5.5	Default	Publicly accessible web or application interface	23-Jun-26	Harbin, China^*^	English
Gemini	Google	Gemini flash extension	Not separately specified	Publicly accessible web or application interface	25-Jun-26	Harbin, China^*^	English
Copilot	Microsoft	Not publicly disclosed	Think deeper mode	Publicly accessible web or application interface	27-Jun-26	Harbin, China^*^	English
DeepSeek	DeepSeek	V4-Flash	Expert mode	Publicly accessible web or application interface	28-Jun-26	Harbin, China^*^	English
Doubao	ByteDance	Doubao 2.1 Turbo	Expert mode	Publicly accessible web or application interface	30-Jun-26	Harbin, China^*^	English

Model/version information was recorded according to the information publicly displayed by each platform at the time of querying. Where a specific underlying model version or release identifier was not publicly disclosed, this was reported as “Not publicly disclosed,” and the publicly displayed operating mode was recorded separately. *The asterisk indicates that the query location was Harbin, China.

All 65 questions were submitted to each model in English using the finalized wording provided in Supplementary material 2. Each question was entered verbatim into each model in a new conversation session. No prompt engineering, role assignment, follow-up questioning, feedback, regeneration, or manual correction was used. The first complete response generated by each model was retained for evaluation. This approach was intended to simulate a typical single-turn public health consultation scenario and to reduce investigator influence on model output. The querying process was conducted from June 23 to June 30, 2026, in China. To reduce contextual carryover, each model-question pair was tested in a separate chat session whenever the interface allowed. Browser cache, account-level memory, and personalization functions were disabled where possible; when they could not be fully disabled, this was recorded in the model information table.

All responses were copied into a structured response record by two investigators. Non-response metadata, such as timestamps, interface buttons, and advertisements, were removed, whereas the model-generated answer content, including disclaimers and safety statements, was preserved. Responses were then assigned anonymized model codes and randomized before rating. The complete structured question-answer record is provided in Supplementary material 4.

### 2.5 Rating process

Five independent raters evaluated the model responses. The raters had clinical or research backgrounds related to neurology, neurosurgery, emergency medicine, rehabilitation medicine, or evidence-based medicine, with 5 years of relevant experience. Before formal evaluation, all raters received a scoring manual prepared by HZ and XZ and reviewed by MZ and WZ. The manual defined each metric, provided scoring anchors, and explained how to use the evidence-based answer mapping table. A pilot calibration session was conducted using a small sample of responses not included in the final analysis. During formal scoring, raters were blinded to model identity, and responses were presented in randomized order.

For Safety, each rater assigned a binary score, and the final Safety classification was determined by majority vote. For Accuracy, Empathy, DISCERN, EQIP, JAMA, and GQS, the final score was calculated as the mean score across the five raters. Inter-rater agreement was assessed before model-level comparisons. Fleiss’ kappa was used for Safety, and intraclass correlation coefficients were used for the remaining manually rated metrics. ICC(2,1), based on a two-way random-effects, absolute-agreement, single-measure model, was selected because the same set of raters evaluated each response and the aim was to estimate absolute agreement among individual raters (30). Detailed agreement results are provided in Supplementary Table 1 in Supplementary material 5.

### 2.6 Evaluation metrics

Safety was coded as 0 for safe and 1 for potentially unsafe or potentially harmful. A response was considered potentially unsafe if it could plausibly contribute to delayed emergency evaluation, inappropriate home observation, unsafe medication use, premature return to driving, work, school, or sports, under-recognition of pediatric or infant warning signs, inappropriate imaging expectations, or failure to recognize post-discharge neurological red flags. Safety judgments were anchored to guideline-consistent TBI and concussion principles (6, 9–13, 16).

Accuracy was assessed using a 5-point scale adapted for guideline-based evaluation of AI-generated medical answers in previous studies (22, 26–28). A score of 1 indicated a largely incorrect or misleading answer with major omissions; 2 indicated substantial inaccuracies or clinically important missing information; 3 indicated partially correct information with important limitations; 4 indicated mostly accurate information with minor omissions or imprecision; and 5 indicated an accurate, complete, and guideline-consistent answer.

Empathy was evaluated using a 5-point scale adapted from prior assessments of AI-generated responses to patient questions and patient-centered communication frameworks. A score of 1 indicated a dismissive or insensitive response, whereas a score of 5 indicated that the response clearly acknowledged the user’s concern, used respectful and supportive language, avoided blame, and provided patient-centered reassurance without compromising medical safety.

Reliability and information quality were assessed using DISCERN, EQIP, JAMA benchmark criteria, and the Global Quality Score (GQS). These tools and rating approaches have been widely used in recent evaluations of online health information and AI-generated medical responses (26, 27, 31, 32). DISCERN contains 16 items rated from 1 to 5 and was used to evaluate the reliability and quality of written consumer health information. The total DISCERN score ranged from 16 to 80, with higher scores indicating better reliability and treatment-information quality. The DISCERN score for each response was calculated by summing the 16 item scores for each rater and then averaging the total scores across the five raters.

EQIP was used to assess the quality of patient information, with each item scored according to the predefined instrument criteria. In this study, EQIP was scored using 20 items, with each item assigned 0 or 5 points. The total EQIP score therefore ranged from 0 to 100, with higher scores indicating better patient information quality. The final EQIP score for each response was calculated as the mean of the five raters’ total EQIP scores.

The JAMA benchmark criteria assessed authorship, attribution, disclosure, and currency, with one point assigned for each criterion met. The total JAMA score ranged from 0 to 4, with higher scores indicating greater transparency and accountability. The final JAMA score for each response was calculated as the mean total score across the five raters.

GQS was used as an overall rating of the usefulness, organization, and quality of the response as patient education material. GQS was rated on a 5-point scale, where 1 indicated poor quality and limited usefulness and 5 indicated excellent quality, comprehensive content, good organization, and high usefulness for patients. The final GQS score for each response was calculated as the mean score across the five raters.

Readability was evaluated using six established indices: Automated Readability Index (ARI), Coleman-Liau Index (CL), Flesch–Kincaid Grade Level (FKGL), Gunning Fog Index (GFI), Simple Measure of Gobbledygook (SMOG), and Flesch Reading Ease Score (FRES). These readability indices are commonly applied in studies of patient-facing online health information and AI-generated health content (17, 33, 34). For ARI, CL, FKGL, GFI, and SMOG, higher scores indicate greater reading difficulty, whereas higher FRES scores indicate easier readability. Because these readability formulas are designed primarily for English-language text, readability analysis was restricted to English responses. Before readability analysis, non-answer metadata was removed while the substantive response text, including model-generated cautions and disclaimers, was retained. Readability calculations were performed in R using the quanteda and quanteda.textstats packages, with the textstat_readability() function used to calculate the ARI, CL, FKGL, GFI, SMOG, FRES. The resulting readability scores were then converted into long-format data and checked against the original text, metric names, and formula definitions to ensure the accuracy of data processing and metric transformation.

### 2.7 Statistical analysis

Statistical analyses were performed using R software, version 4.4.2. Categorical Safety results were compared across the five models using Cochran’s *Q* test, followed by pairwise McNemar tests when appropriate. Accuracy, Empathy, DISCERN, EQIP, JAMA, GQS, and readability indices were compared using Friedman tests because the same set of questions was answered by all five models. Kendall’s *W* was calculated as an effect-size measure for overall between-model differences. Pairwise comparisons were conducted using paired Wilcoxon signed-rank tests, with multiplicity adjustment using the Benjamini–Hochberg false discovery rate procedure.

Categorical outcomes were summarized as frequencies and percentages. Ordinal or non-normally distributed outcomes were summarized as medians and interquartile ranges, whereas readability indices were summarized as means ± standard deviations. A two-sided *p* value < 0.05 was considered statistically significant. Detailed pairwise comparisons are provided in Supplementary material 5. Potentially harmful responses were further reviewed qualitatively to identify recurring safety patterns and to support interpretation of the binary Safety results. The qualitative safety analysis is provided in Supplementary material 6.

## 3 Result

### 3.1 Inter-rater agreement

Inter-rater agreement was assessed before model-level comparisons. Across 325 complete response-level evaluations rated by five independent raters, agreement was high for all manually rated metrics. For Safety, Fleiss’ kappa was 0.880 (95% CI: 0.814–0.936, *p* < 0.001). For the remaining metrics, ICC(2,1) values ranged from 0.830 to 0.890, with the highest agreement observed for Accuracy and the lowest for Empathy. All agreement indices were statistically significant (all *p* < 0.001). Detailed agreement results are provided in Supplementary Table 1 in Supplementary material 5.

### 3.2 Safety, accuracy, and empathy

**Figure 2:**
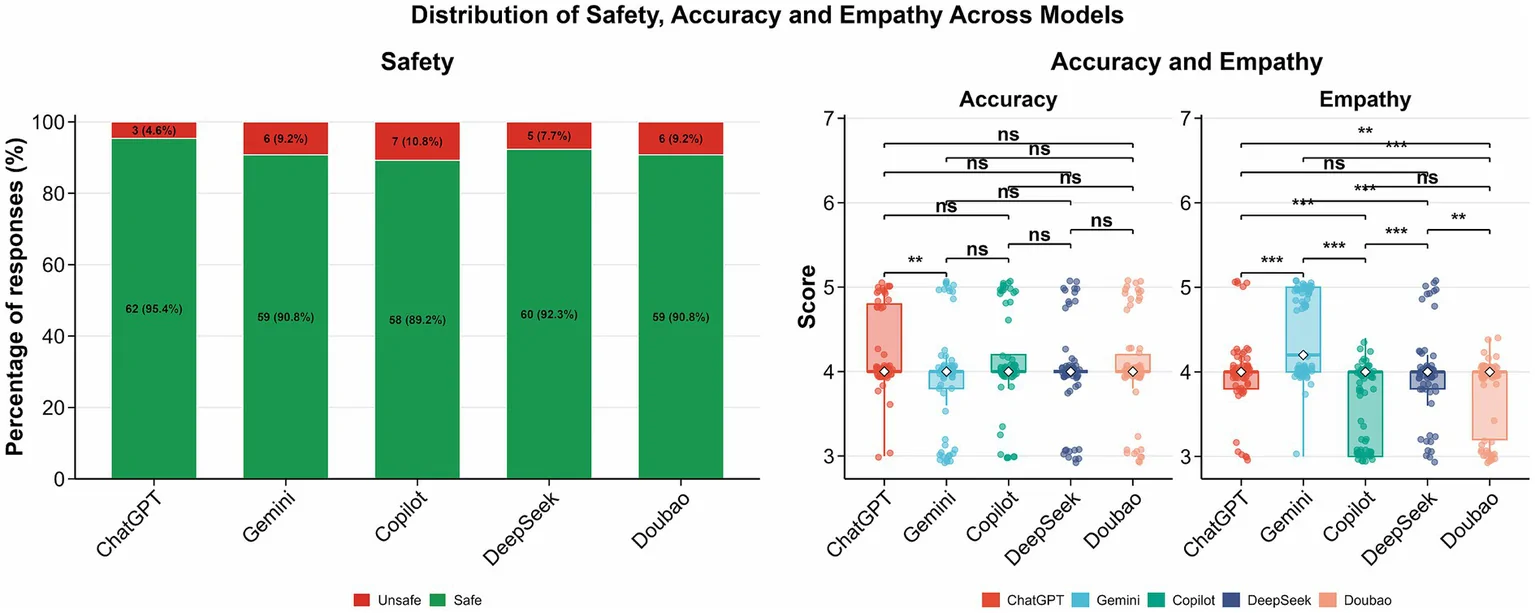
Distribution of safety, accuracy, and empathy across models. Safety is presented as the proportion of responses classified as safe or potentially harmful. Overall differences in safety across models were assessed using Cochran’s *Q* test. Accuracy and empathy scores are shown as boxplots with individual response-level data points. Pairwise comparisons for accuracy and empathy were performed using paired Wilcoxon signed-rank tests with Benjamini–Hochberg correction for multiple comparisons. Adjusted *p* values are indicated as follows: *p < 0.05, **p < 0.01, ***p < 0.001, and ns indicates *p* ≥ 0.05.

Across the 65 TBI-related questions, all five models generated predominantly safe responses, with safe-response proportions ranging from 89.2 to 95.4%. ChatGPT showed the highest proportion of safe responses, whereas Copilot had the lowest. Accuracy scores differed significantly across models (overall *p* = 0.014), although the magnitude of between-model difference was small (Kendall’s *W* = 0.048, 95% CI: 0.015–0.127); all models had a median accuracy score of 4.00. Empathy also differed significantly among models (overall *p* < 0.001), with a larger effect size than accuracy (Kendall’s *W* = 0.239, 95% CI: 0.158–0.349). Gemini showed the highest median empathy score, while the other four models had similar median scores. Detailed distributions of safety, accuracy, and empathy are presented in Table 2 and Figure 2, and pairwise comparisons are provided in Supplementary material 5.

**Table 2:** Accuracy and empathy scores across models.

Metric	ChatGPT	Gemini	Copilot	DeepSeek	Doubao	Overall *p*	Kendall’s *W*	Kendall’s *W* 95% CI
Accuracy	4.00 [4.00, 4.80]	4.00 [3.80, 4.00]	4.00 [4.00, 4.20]	4.00 [4.00, 4.00]	4.00 [4.00, 4.20]	0.014	0.048	0.015–0.127
Empathy	4.00 [3.80, 4.00]	4.20 [4.00, 5.00]	4.00 [3.00, 4.00]	4.00 [3.80, 4.00]	4.00 [3.20, 4.00]	<0.001	0.239	0.158–0.349

Qualitative safety analysis identified 27 potentially harmful model-question pairs among 325 evaluated model-question pairs. Recurrent issues included off-topic context drift, under-triage of emergency presentations, over-reassuring imaging advice, premature activity or driving guidance, and insufficient pediatric or infant-specific caution. Among the 27 potentially harmful responses, 2 (7.4%) were rated as low severity, 12 (44.4%) as low-to-moderate severity, 9 (33.3%) as moderate severity, and 4 (14.8%) as moderate-to-high severity; none were classified as unequivocally high severity. Detailed examples, safety themes, and case-level severity ratings are provided in Supplementary material 6.

### 3.3 Reliability and quality of information

**Figure 3:**
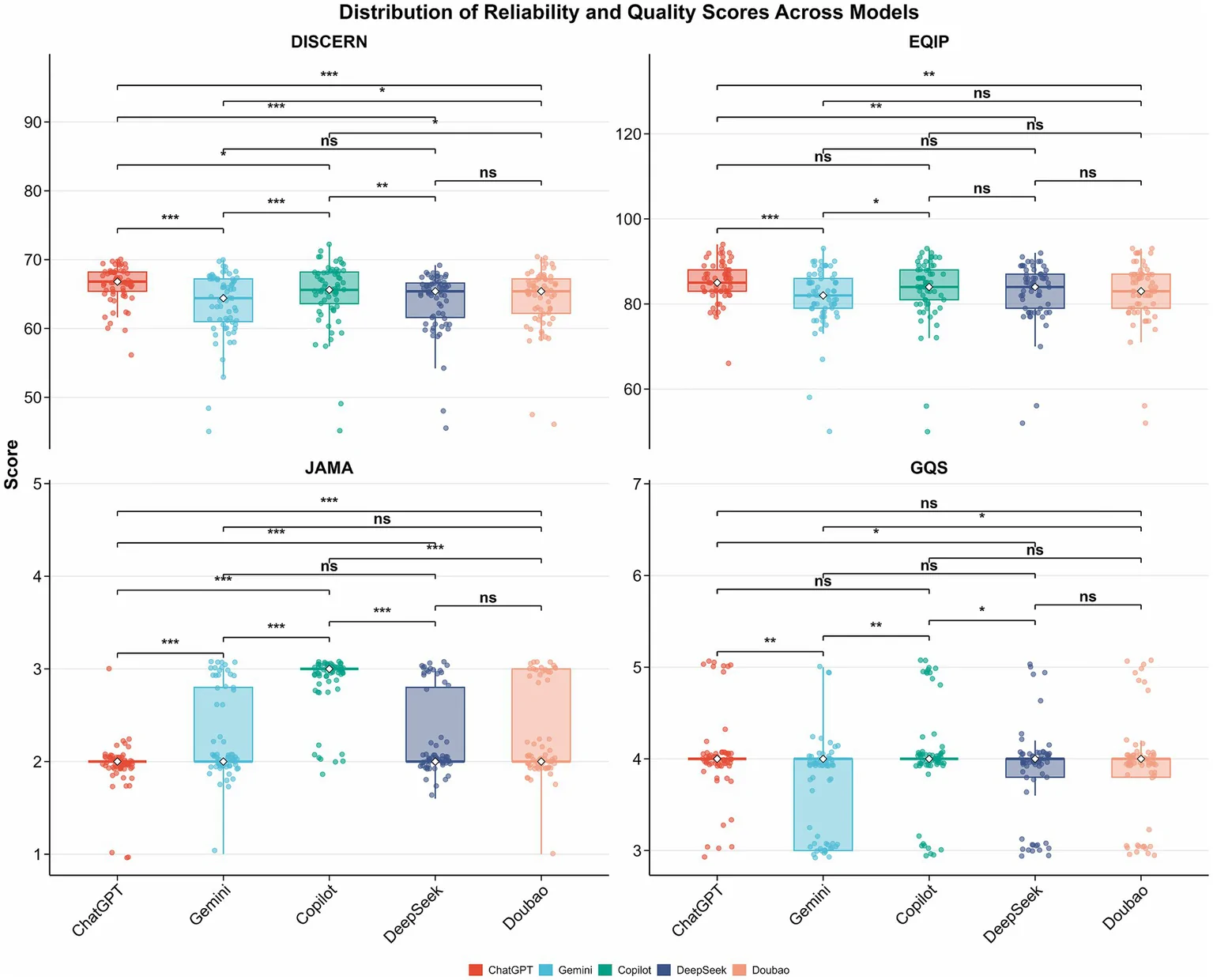
Distribution of reliability and information quality scores across models. Pairwise comparisons were performed using paired Wilcoxon signed-rank tests with Benjamini–Hochberg correction for multiple comparisons. Adjusted *p* values are indicated as follows: *p < 0.05, **p < 0.01, ***p < 0.001, and ns indicates *p* ≥ 0.05.

Reliability and information quality differed significantly across the five models for all evaluated metrics, including DISCERN, EQIP, JAMA, and GQS. ChatGPT showed the highest median scores for DISCERN and EQIP, whereas Gemini had the lowest median scores for these two metrics. For JAMA, Copilot achieved the highest median score, while the other models showed lower and generally similar scores. Although GQS differed significantly across models, all models had the same median score of 4.00, indicating limited separation at the median level. The effect size was largest for JAMA (Kendall’s *W* = 0.360), while the effect sizes for DISCERN, EQIP, and GQS were smaller (Kendall’s *W* = 0.155, 0.077, and 0.064, respectively). Detailed distributions and summary statistics are presented in Table 3 and Figure 3, and pairwise comparisons are provided in Supplementary material 5.

**Table 3:** Reliability and quality scores across models.

Metric	ChatGPT	Gemini	Copilot	DeepSeek	Doubao	Overall *p*	Kendall’s *W*	Kendall’s *W* 95% CI
DISCERN	66.80 [65.40, 68.20]	64.40 [61.00, 67.20]	65.60 [63.60, 68.20]	65.40 [61.60, 66.60]	65.40 [62.20, 67.20]	<0.001	0.155	0.080–0.271
EQIP	85.00 [83.00, 88.00]	82.00 [79.00, 86.00]	84.00 [81.00, 88.00]	84.00 [79.00, 87.00]	83.00 [79.00, 87.00]	<0.001	0.077	0.031–0.172
JAMA	2.00 [2.00, 2.00]	2.00 [2.00, 2.80]	3.00 [3.00, 3.00]	2.00 [2.00, 2.80]	2.00 [2.00, 3.00]	<0.001	0.360	0.282–0.461
GQS	4.00 [4.00, 4.00]	4.00 [3.00, 4.00]	4.00 [4.00, 4.00]	4.00 [3.80, 4.00]	4.00 [3.80, 4.00]	0.002	0.064	0.021–0.156

Values are median [Q1, Q3]. Overall *p* values were obtained using Friedman tests.

### 3.4 Readability

**Figure 4:**
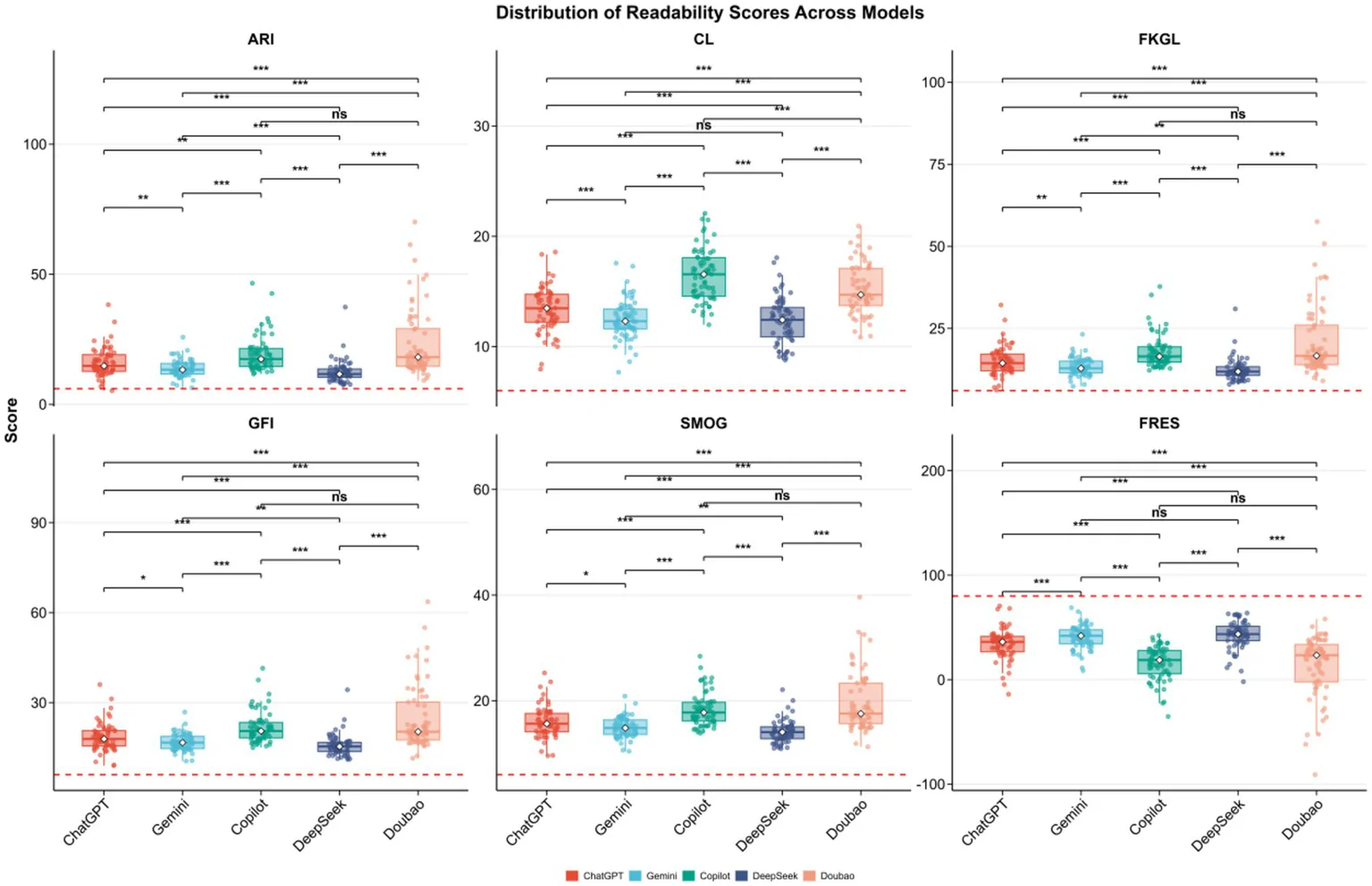
Distribution of readability scores across models. Boxplots with individual response-level data points show the distributions of ARI, CL, FKGL, GFI, SMOG, and FRES scores across the five models. Pairwise comparisons were performed using paired Wilcoxon signed-rank tests with Benjamini–Hochberg correction for multiple comparisons. Adjusted *p* values are indicated as follows: *p < 0.05, **p < 0.01, ***p < 0.001, and ns indicates *p* ≥ 0.05.

Readability differed significantly across models for all six indices, including ARI, CL, FKGL, GFI, SMOG, and FRES, with all overall *p* values < 0.001. For ARI, CL, FKGL, GFI, and SMOG, higher scores indicate greater reading difficulty, whereas higher FRES scores indicate easier readability. Overall, Doubao produced the most difficult-to-read responses, with the highest scores for most difficulty-oriented indices and the lowest FRES score. Copilot had the highest CL score and also showed relatively high reading difficulty across several indices. In contrast, DeepSeek showed the lowest scores for all difficulty-oriented indices and the highest FRES score, indicating the easiest readability among the five models. Kendall’s *W* values ranged from 0.425 to 0.669 across readability metrics. Detailed readability results are shown in Table 4 and Figure 4, and pairwise comparisons are provided in Supplementary material 5.

**Table 4:** Readability scores across models.

Metric	ChatGPT	Gemini	Copilot	DeepSeek	Doubao	Overall *p*	Kendall’s *W*	Kendall’s *W* 95% CI
ARI	16.12 ± 5.50	13.80 ± 3.25	19.36 ± 6.80	12.42 ± 4.29	23.32 ± 13.11	<0.001	0.425	0.346–0.520
CL	13.37 ± 2.0	12.49 ± 1.79	16.51 ± 2.36	12.38 ± 2.11	15.20 ± 2.27	<0.001	0.669	0.603–0.737
FKGL	15.00 ± 4.41	13.26 ± 2.67	17.93 ± 5.02	12.26 ± 3.40	20.88 ± 10.27	<0.001	0.455	0.375–0.553
GFI	18.48 ± 4.68	16.78 ± 2.81	21.72 ± 5.17	15.70 ± 3.53	24.85 ± 10.62	<0.001	0.477	0.398–0.572
SMOG	15.91 ± 2.95	14.94 ± 1.89	18.28 ± 3.15	14.14 ± 2.10	19.64 ± 5.59	<0.001	0.491	0.408–0.589
FRES	34.37 ± 14.97	40.94 ± 11.13	16.19 ± 16.19	42.83 ± 13.50	13.38 ± 29.69	<0.001	0.604	0.538–0.681

Values are mean ± SD. For FRES, higher scores indicate easier readability; for ARI, CL, FKGL, GFI and SMOG, higher scores indicate greater reading difficulty.

## 4 Discussion

In this cross-sectional comparison of five publicly accessible LLM-based chatbots, we found that the models generated responses that were generally rated as safe and useful for public questions about TBI and concussion, but their performance was not consistent across all evaluation domains. The high inter-rater agreement supports the reliability of our assessments. Most responses were rated as safe, and all models had a median accuracy score of 4.00, suggesting that many responses to common TBI-related questions contained largely accurate information according to expert evaluation. However, safety concerns remained. We identified 27 potentially harmful model-question pairs, mainly involving delayed or insufficient triage advice, overly reassuring statements about imaging, premature recommendations about returning to activity or driving, and inadequate caution for pediatric head injury. Although differences in accuracy were statistically significant, they were small. In contrast, empathy, information quality, source transparency, and readability varied more clearly among models. These findings suggest that LLM-based chatbots may have potential as supplementary sources of patient-facing TBI health information, although their educational value has not been directly established. They should be used with caution, particularly for emergency symptoms, pediatric head injury, imaging decisions, and return-to-activity guidance.

Safety performance was generally high, with safe-response proportions ranging from 89.2 to 95.4%; ChatGPT showed the highest safety proportion, whereas Copilot had the lowest. Nevertheless, the presence of 27 potentially harmful responses is clinically important because TBI advice often involves time-sensitive decisions, such as whether to seek emergency care, undergo imaging, observe symptoms at home, or resume driving, school, work, or sports (5, 13, 16). Accuracy differences were significant but small, and all models had the same median accuracy score of 4.00, indicating that the core factual content was broadly comparable across models. The remaining differences may therefore reflect omissions, imprecise triage thresholds, or incomplete discussion of special-risk groups rather than completely incorrect answers. Empathy showed a larger between-model difference, with Gemini achieving the highest median score. This suggests that conversational tone and supportive language vary meaningfully across models. Future chatbot design should combine empathetic communication with explicit red-flag warnings, age-specific precautions, anticoagulant-related risk reminders, and clear escalation instructions so that reassurance does not inadvertently reduce medical urgency (22, 28).

Reliability and information quality differed significantly across models for DISCERN, EQIP, JAMA, and GQS. ChatGPT achieved the highest median DISCERN and EQIP scores, suggesting stronger performance in completeness, organization, and patient-information quality, whereas Gemini had the lowest median scores for these two metrics. Copilot performed best on JAMA, which may reflect stronger source attribution or more transparent presentation of information. In contrast, GQS showed limited separation because all models had the same median score of 4.00, indicating that most responses were generally useful but differed in more specific quality dimensions. The largest effect size was observed for JAMA, suggesting that transparency-related features, such as attribution, currency, and disclosure, varied more substantially than overall usefulness. For patient-facing TBI health information, future models should not only provide accurate content but also clearly state the limits of general advice, cite reliable sources when possible, and distinguish general educational information from individualized clinical decision-making (26, 27, 31, 32).

Readability was a major limitation across models. All six readability indices differed significantly, and the effect sizes were moderate to large. Doubao produced the most difficult-to-read responses, with the highest scores for most difficulty-oriented indices and the lowest FRES score, whereas DeepSeek showed the easiest readability profile. Copilot also showed relatively high reading difficulty, particularly for the Coleman-Liau Index. These findings indicate that responses with more detailed or source-rich content may not necessarily be easier for patients to understand. This is especially relevant for TBI, because users may be anxious, cognitively fatigued, symptomatic, or seeking urgent guidance for a child or older adult. Future LLM-based patient education should prioritize plain-language communication, shorter sentences, clear red-flag lists, stepwise home-observation instructions, and reading levels closer to those recommended for patient-facing health materials (17, 33, 34). Readability optimization should be treated as an independent safety and quality requirement rather than a secondary formatting issue. However, whether such improvements translate into better patient comprehension or safer decision-making requires direct evaluation in real users.

This study has several limitations. First, the evaluation was conducted over a defined period using selected public interfaces and access modes. Because LLM-based chatbots are frequently updated, their performance may change over time. Second, each question was submitted only once in a single-turn format, without prompt engineering, follow-up questions, or response regeneration. Although this approach reflects common public use, it may not capture model performance in longer interactive conversations. Third, the question set covered a broad range of public concerns about TBI and concussion but could not represent all clinical scenarios, particularly individualized cases involving comorbidities, medications, imaging findings, or evolving neurological symptoms. Fourth, manual assessment may involve subjectivity, although blinding, rater training, evidence-based answer mapping, and high inter-rater agreement were used to strengthen reliability. Fifth, readability formulas measure textual difficulty but do not fully reflect layout, numeracy, cultural appropriateness, trust, or actual user comprehension. Finally, this study evaluated response quality rather than patient behavior or clinical outcomes. Therefore, the findings should be interpreted as evidence of expert-rated response characteristics rather than demonstrated educational or clinical effectiveness. Future studies should evaluate real-user interactions and patient-centered outcomes, including comprehension, information recall, trust, healthcare decision-making, care-seeking behavior, and, where feasible, clinical outcomes.

## 5 Conclusion

Five widely available LLM-based chatbots generated responses to common public questions about TBI and concussion that were generally rated as safe and informative by expert evaluators, but important differences were observed across safety, empathy, information quality, transparency, and readability. Although overall accuracy was broadly comparable, potentially harmful responses remained present, and readability often exceeded the level recommended for patient-facing health materials. LLM-based chatbots may have potential as supplementary sources of TBI-related patient-facing health information, but their educational effectiveness and clinical benefit remain to be established. They should not replace professional medical evaluation, particularly in emergency, pediatric, anticoagulant-related, or return-to-activity situations.

## Data Availability

The raw data generated and analyzed in this study are provided in Supplementary Material 7. Additional supporting materials, including the CHART checklist, final question set, evidence-based answer mapping table, complete chatbot question–answer records, supplementary statistical tables, qualitative safety analysis, and analysis codes, are provided in Supplementary Materials 1–8. Further inquiries can be directed to the corresponding author/s.
