# Supplementary material for: Safety, accuracy, empathy, information quality, and readability of publicly accessible LLM-based chatbots for traumatic brain injury and concussion questions: a cross-sectional comparative study
Source: Front Public Health. 2026 Sep 1;14:1926980. doi: 10.3389/fpubh.2026.1926980 (PMC13574619; doi:10.3389/fpubh.2026.1926980)
Supplement: Supplementary file 1 [file Data_Sheet_1.zip › Supplementary Material 1.CHART Checklist.docx]

**Supplementary Material 1.CHART Checklist**

| **Heading** | **No** | **Chart checklist item** | **Page No** |
| --- | --- | --- | --- |
| **Title and abstract** |  |  |  |
| Title | 1a | State that the study is assessing one or more generative AI-driven chatbots for clinical evidence or health advice. | P 1 |
| Abstract/summary | 1b | Apply a structured format, if applicable. | P 1 |
| **Introduction** |  |  |  |
| Background | 2a | State the scientific background, rationale, and healthcare context for evaluating the generative AI-driven chatbot(s), referencing relevant literature when applicable. | pP 1–3 |
|  | 2b | State the aims and research questions including the target audience, intervention, comparator(s), and outcome(s). | P 3 |
| **Methods** |  |  |  |
| Model identifiers | 3a | State the name and version identifier(s) of the generative AI model(s) and chatbot(s) under evaluation, as well as their date of release or last update. | P 5; Tbl. 1, pP 20–21 |
|  | 3b | State whether the generative AI model(s) and chatbot(s) are open-source or closed-source/proprietary. |  |
| Model details | 4a | State whether the generative AI model was a base model or a novel base model, tuned model, or fine-tuned model. |  |
|  | 4b | If a base model is used, cite its development in sufficient detail to identify the model. |  |
|  | 4c | If a novel base model, tuned model, or fine-tuned model is used, describe the pre- and/or post-implementation/deployment data and parameters. |  |
| Prompt engineering | 5a | Describe the evolution of study prompt development. | P 5 |
|  | 5ai | Describe the sources of prompts. | P 3–4 |
|  | 5aii | State the number and characteristics of the individual(s) involved in prompt engineering. | P 3–4 |
|  | 5aiii | Provide details of any patient and public involvement during prompt engineering. | P 3 |
|  | 5b | Provide study prompts. | P 4; Suppl. 2 |
| Query strategy | 6a | State route of access to generative AI model. | P 5; Tbl. 1, pP 20–21 |
|  | 6b | State the date(s) and location(s) of queries for the generative AI-driven chatbot(s) including the day, month, and year as well as city and country. | P 5; Tbl. 1, pP 20–21 |
|  | 6c | Describe whether prompts were input into separate chat session(s). | P 5 |
|  | 6d | Provide all generative AI-driven chatbot output/responses | P 5–6; Suppl. 4 |
| Performance evaluation | 7a | Define the ground truth or reference standard used to define successful generative AI-driven chatbot performance. | P 5 |
|  | 7b | Describe the process undertaken for generative AI-driven chatbot performance evaluation. | P 6–8 |
|  | 7bi | State the number and characteristics of team members involved in performance evaluation. | P 6 |
|  | 7bii | Provide details of any patients and public involvement during the evaluation process. | P 3 |
|  | 7biii | State whether evaluators were blinded to the identity of the generative AI-driven chatbot(s) under assessment. | P 6 |
| Sample size | 8 | Report how the sample size was determined. | P 4–5 |
| Data analysis | 9a | Describe statistical analysis methods, including any evaluation of reproducibility of generative AI-driven chatbot responses. | P 8 |
|  | 9ai | Report the measures used for performance evaluation. | P 6–8 |
| **Results** |  |  |  |
|  | 10a | Report the performance evaluation undertaken including the alignment between generative AI-driven chatbot output and ground truth or reference standard using quantitative or mixed methods approaches as applicable. | P 9–10; Tables 2–4, P 21–22; Figs. 2–4, P 19 |
|  | 10b | For responses deviating from the ground truth or reference standard, state the nature of the difference(s). | P 9; Suppl. 6 |
|  | 10c | Report the evaluation for potentially harmful, biased, or misleading responses. | P 9–12; Suppl. 6 |
| **Discussion** |  |  |  |
|  | 11a | Interpret study findings in the context of relevant evidence. | P 10–12 |
|  | 11b | Describe the strengths and limitations of the study. | P 10–12 |
|  | 11c | Describe the potential implications for practice, education, policy, regulation, and research. | P 10–13 |
| **Open science** |  |  |  |
| Disclosures | 12a | Report any relevant conflicts of interest for all authors. | P 13 |
| Funding | 12b | Report sources of funding and their role in the conduct and reporting of the study. | P 13 |
| Ethics | 12c | Describe the process undertaken for ethical approval. | P 3, 13 |
|  | 12ci | Describe the measures taken to safeguard data privacy of patient health information, as applicable. | P 3, 13 |
|  | 12cii | State whether permission/licensing was obtained for the use of original, copyrighted data. |  |
| Protocol | 12d | Provide a study protocol. |  |
| Data availability | 12e | State where study data, code repository, and model parameters can be accessed. | P 13 |

Footer Note: AI, artificial intelligence.
