# Supplementary material for: Safety, accuracy, empathy, information quality, and readability of publicly accessible LLM-based chatbots for traumatic brain injury and concussion questions: a cross-sectional comparative study
Source: Front Public Health. 2026 Sep 1;14:1926980. doi: 10.3389/fpubh.2026.1926980 (PMC13574619; doi:10.3389/fpubh.2026.1926980)
Supplement: Supplementary file 1 [file Data_Sheet_1.zip › Supplementary Material 5. Supplementary tables.docx]

**Supplementary Material 5. Supplementary tables**

## Table S1. Inter-rater agreement

| **Metric** | **Agreement index** | **Estimate** | **95% CI** | **P value** | **Complete items** | **No. of raters** | **Note** |
| --- | --- | --- | --- | --- | --- | --- | --- |
| Safety | Fleiss' kappa | 0.880 | 0.814-0.936 | <0.001 | 325 | 5 | Primary agreement index for Safety; 95% CI based on 3000 nonparametric bootstrap resamples. A negative lower CI may occur when unsafe outcomes are extremely sparse because kappa can theoretically be below 0; this does not by itself indicate a coding error. |
| Accuracy | ICC(2,1) | 0.890 | 0.872-0.907 | <0.001 | 325 | 5 | Two-way random-effects, absolute-agreement, single-measure ICC. |
| Empathy | ICC(2,1) | 0.830 | 0.803-0.854 | <0.001 | 325 | 5 | Two-way random-effects, absolute-agreement, single-measure ICC. |
| DISCERN | ICC(2,1) | 0.885 | 0.866-0.903 | <0.001 | 325 | 5 | Two-way random-effects, absolute-agreement, single-measure ICC. |
| EQIP | ICC(2,1) | 0.878 | 0.858-0.896 | <0.001 | 325 | 5 | Two-way random-effects, absolute-agreement, single-measure ICC. |
| JAMA | ICC(2,1) | 0.856 | 0.834-0.877 | <0.001 | 325 | 5 | Two-way random-effects, absolute-agreement, single-measure ICC. |
| GQS | ICC(2,1) | 0.884 | 0.865-0.901 | <0.001 | 325 | 5 | Two-way random-effects, absolute-agreement, single-measure ICC. |

Note: Safety was assessed using Fleiss' kappa. Accuracy, Empathy, DISCERN, EQIP, JAMA and GQS were assessed using ICC(2,1).

## Table S2. Pairwise comparisons of safety

| **Comparison** | **N pairs** | **Safe rate difference, %** | **Safe rate difference 95% CI** | **Safe to unsafe, n** | **Unsafe to safe, n** | **Matched OR** | **Matched OR 95% CI** | **Adjusted P** | **Note** |
| --- | --- | --- | --- | --- | --- | --- | --- | --- | --- |
| ChatGPT vs Gemini | 65 | 4.62 | -1.54-12.31 | 4 | 1 | 4.00 | 0.45-35.79 | 1.000 | Pairwise McNemar test |
| ChatGPT vs Copilot | 65 | 6.15 | -3.08-15.38 | 7 | 3 | 2.33 | 0.60-9.02 | 1.000 | Pairwise McNemar test |
| ChatGPT vs DeepSeek | 65 | 3.08 | -4.62-10.77 | 4 | 2 | 2.00 | 0.37-10.92 | 1.000 | Pairwise McNemar test |
| ChatGPT vs Doubao | 65 | 4.62 | -1.54-12.31 | 4 | 1 | 4.00 | 0.45-35.79 | 1.000 | Pairwise McNemar test |
| Gemini vs Copilot | 65 | 1.54 | -6.15-9.23 | 4 | 3 | 1.33 | 0.30-5.96 | 1.000 | Pairwise McNemar test |
| Gemini vs DeepSeek | 65 | -1.54 | -6.15-3.08 | 1 | 2 | 0.50 | 0.05-5.51 | 1.000 | Pairwise McNemar test |
| Gemini vs Doubao | 65 | 0.00 | -6.15-6.15 | 2 | 2 | 1.00 | 0.14-7.10 | 1.000 | Pairwise McNemar test |
| Copilot vs DeepSeek | 65 | -3.08 | -10.77-4.62 | 2 | 4 | 0.50 | 0.09-2.73 | 1.000 | Pairwise McNemar test |
| Copilot vs Doubao | 65 | -1.54 | -10.77-6.15 | 4 | 5 | 0.80 | 0.21-2.98 | 1.000 | Pairwise McNemar test |
| DeepSeek vs Doubao | 65 | 1.54 | -4.62-7.69 | 3 | 2 | 1.50 | 0.25-8.98 | 1.000 | Pairwise McNemar test |

Note: Pairwise comparisons were performed using McNemar tests with Benjamini-Hochberg correction. Safe-rate differences and their bootstrap 95% CIs are reported.

## Table S3. Significant pairwise comparisons for non-binary outcomes

| **Metric** | **Comparison** | **N pairs** | **Mean difference** | **Mean difference 95% CI** | **Median difference** | **Median difference 95% CI** | **Rank-biserial correlation** | **Rank-biserial 95% CI** | **Adjusted P** |
| --- | --- | --- | --- | --- | --- | --- | --- | --- | --- |
| Accuracy | ChatGPT vs Gemini | 65 | 0.27 | 0.14-0.40 | 0.00 | 0.00-0.20 | 0.684 | 0.396-0.907 | 0.003 |
| Empathy | ChatGPT vs Gemini | 65 | -0.45 | -0.59--0.30 | -0.20 | -0.80-0.00 | -0.791 | -0.942--0.564 | <0.001 |
| Empathy | ChatGPT vs Copilot | 65 | 0.35 | 0.21-0.50 | 0.20 | 0.00-0.20 | 0.644 | 0.398-0.834 | <0.001 |
| Empathy | ChatGPT vs Doubao | 65 | 0.23 | 0.10-0.36 | 0.00 | 0.00-0.20 | 0.546 | 0.236-0.801 | 0.003 |
| Empathy | Gemini vs Copilot | 65 | 0.80 | 0.64-0.96 | 1.00 | 0.80-1.00 | 0.967 | 0.909-0.995 | <0.001 |
| Empathy | Gemini vs DeepSeek | 65 | 0.46 | 0.30-0.62 | 0.60 | 0.00-1.00 | 0.757 | 0.537-0.910 | <0.001 |
| Empathy | Gemini vs Doubao | 65 | 0.68 | 0.53-0.83 | 0.80 | 0.40-1.00 | 0.980 | 0.930-1.000 | <0.001 |
| Empathy | Copilot vs DeepSeek | 65 | -0.34 | -0.47--0.21 | -0.20 | -0.40-0.00 | -0.698 | -0.887--0.463 | <0.001 |
| Empathy | DeepSeek vs Doubao | 65 | 0.22 | 0.09-0.38 | 0.00 | 0.00-0.20 | 0.483 | 0.165-0.736 | 0.008 |
| DISCERN | ChatGPT vs Gemini | 65 | 2.87 | 1.95-3.79 | 2.40 | 1.40-3.60 | 0.723 | 0.528-0.870 | <0.001 |
| DISCERN | ChatGPT vs Copilot | 65 | 1.19 | 0.36-2.10 | 0.80 | 0.00-1.80 | 0.347 | 0.060-0.597 | 0.027 |
| DISCERN | ChatGPT vs DeepSeek | 65 | 2.42 | 1.50-3.35 | 2.00 | 0.80-3.20 | 0.662 | 0.464-0.835 | <0.001 |
| DISCERN | ChatGPT vs Doubao | 65 | 2.10 | 1.30-2.96 | 1.80 | 1.00-3.00 | 0.643 | 0.428-0.828 | <0.001 |
| DISCERN | Gemini vs Copilot | 65 | -1.68 | -2.47--0.89 | -1.60 | -2.40--1.00 | -0.570 | -0.786--0.319 | <0.001 |
| DISCERN | Gemini vs Doubao | 65 | -0.77 | -1.50--0.08 | -0.80 | -1.80-0.20 | -0.304 | -0.562--0.039 | 0.043 |
| DISCERN | Copilot vs DeepSeek | 65 | 1.23 | 0.34-2.12 | 1.60 | 1.00-2.20 | 0.443 | 0.171-0.689 | 0.005 |
| DISCERN | Copilot vs Doubao | 65 | 0.91 | -0.04-1.82 | 1.00 | 0.20-1.60 | 0.344 | 0.074-0.609 | 0.027 |
| EQIP | ChatGPT vs Gemini | 65 | 3.54 | 2.12-5.03 | 4.00 | 1.00-5.00 | 0.632 | 0.408-0.808 | <0.001 |
| EQIP | ChatGPT vs DeepSeek | 65 | 2.28 | 0.92-3.80 | 2.00 | 0.00-3.00 | 0.473 | 0.195-0.709 | 0.010 |
| EQIP | ChatGPT vs Doubao | 65 | 2.31 | 0.91-3.72 | 2.00 | 1.00-4.00 | 0.453 | 0.172-0.695 | 0.010 |
| EQIP | Gemini vs Copilot | 65 | -1.80 | -3.00--0.65 | -2.00 | -4.00--1.00 | -0.382 | -0.634--0.118 | 0.022 |
| JAMA | ChatGPT vs Gemini | 65 | -0.31 | -0.46--0.18 | 0.00 | -0.20-0.00 | -0.715 | -0.905--0.449 | <0.001 |
| JAMA | ChatGPT vs Copilot | 65 | -0.89 | -0.98--0.78 | -1.00 | -1.00--1.00 | -0.998 | -1.000--0.989 | <0.001 |
| JAMA | ChatGPT vs DeepSeek | 65 | -0.30 | -0.44--0.18 | 0.00 | -0.20-0.00 | -0.782 | -0.940--0.523 | <0.001 |
| JAMA | ChatGPT vs Doubao | 65 | -0.31 | -0.46--0.17 | 0.00 | -0.20-0.00 | -0.668 | -0.867--0.392 | <0.001 |
| JAMA | Gemini vs Copilot | 65 | -0.58 | -0.71--0.45 | -0.80 | -1.00--0.20 | -0.934 | -1.000--0.805 | <0.001 |
| JAMA | Copilot vs DeepSeek | 65 | 0.59 | 0.43-0.74 | 1.00 | 0.80-1.00 | 0.792 | 0.594-0.946 | <0.001 |
| JAMA | Copilot vs Doubao | 65 | 0.58 | 0.44-0.70 | 0.80 | 0.60-1.00 | 0.886 | 0.729-0.998 | <0.001 |
| GQS | ChatGPT vs Gemini | 65 | 0.31 | 0.16-0.46 | 0.00 | 0.00-0.20 | 0.637 | 0.362-0.862 | 0.004 |
| GQS | ChatGPT vs DeepSeek | 65 | 0.17 | 0.04-0.30 | 0.00 | 0.00-0.00 | 0.486 | 0.138-0.788 | 0.036 |
| GQS | Gemini vs Copilot | 65 | -0.31 | -0.48--0.14 | 0.00 | -0.20-0.00 | -0.584 | -0.841--0.268 | 0.006 |
| GQS | Gemini vs Doubao | 65 | -0.18 | -0.33--0.05 | 0.00 | 0.00-0.00 | -0.447 | -0.749--0.088 | 0.046 |
| GQS | Copilot vs DeepSeek | 65 | 0.17 | 0.02-0.32 | 0.00 | 0.00-0.00 | 0.466 | 0.091-0.797 | 0.046 |
| ARI | ChatGPT vs Gemini | 65 | 2.32 | 1.04-3.71 | 1.30 | 0.25-2.54 | 0.406 | 0.147-0.637 | 0.005 |
| ARI | ChatGPT vs Copilot | 65 | -3.24 | -5.23--1.37 | -2.50 | -4.37-0.17 | -0.439 | -0.657--0.179 | 0.003 |
| ARI | ChatGPT vs DeepSeek | 65 | 3.71 | 2.58-4.91 | 2.63 | 1.37-4.87 | 0.766 | 0.579-0.905 | <0.001 |
| ARI | ChatGPT vs Doubao | 65 | -7.20 | -10.32--4.40 | -4.14 | -6.34--1.41 | -0.621 | -0.799--0.406 | <0.001 |
| ARI | Gemini vs Copilot | 65 | -5.56 | -7.19--4.02 | -4.69 | -6.70--2.69 | -0.855 | -0.967--0.703 | <0.001 |
| ARI | Gemini vs DeepSeek | 65 | 1.39 | 0.20-2.46 | 1.49 | 0.34-2.34 | 0.496 | 0.243-0.711 | <0.001 |
| ARI | Gemini vs Doubao | 65 | -9.52 | -12.94--6.44 | -4.45 | -7.03--2.92 | -0.842 | -0.948--0.698 | <0.001 |
| ARI | Copilot vs DeepSeek | 65 | 6.94 | 5.23-8.75 | 5.14 | 4.40-7.66 | 0.888 | 0.767-0.980 | <0.001 |
| ARI | DeepSeek vs Doubao | 65 | -10.91 | -14.19--8.07 | -6.13 | -8.60--4.50 | -0.978 | -1.000--0.919 | <0.001 |
| CL | ChatGPT vs Gemini | 65 | 0.88 | 0.51-1.24 | 0.99 | 0.59-1.52 | 0.594 | 0.375-0.791 | <0.001 |
| CL | ChatGPT vs Copilot | 65 | -3.14 | -3.63--2.66 | -2.91 | -3.80--2.34 | -0.999 | -1.000--0.994 | <0.001 |
| CL | ChatGPT vs DeepSeek | 65 | 0.99 | 0.60-1.36 | 1.10 | 0.62-1.52 | 0.661 | 0.437-0.859 | <0.001 |
| CL | ChatGPT vs Doubao | 65 | -1.84 | -2.33--1.38 | -2.00 | -2.41--1.26 | -0.824 | -0.932--0.678 | <0.001 |
| CL | Gemini vs Copilot | 65 | -4.02 | -4.52--3.51 | -3.94 | -4.59--3.40 | -1.000 | -1.000--1.000 | <0.001 |
| CL | Gemini vs Doubao | 65 | -2.71 | -3.21--2.21 | -2.74 | -3.19--2.27 | -0.956 | -0.993--0.892 | <0.001 |
| CL | Copilot vs DeepSeek | 65 | 4.13 | 3.67-4.61 | 4.26 | 3.82-4.95 | 0.999 | 0.994-1.000 | <0.001 |
| CL | Copilot vs Doubao | 65 | 1.30 | 0.80-1.79 | 1.27 | 0.81-2.00 | 0.647 | 0.442-0.840 | <0.001 |
| CL | DeepSeek vs Doubao | 65 | -2.82 | -3.19--2.43 | -2.93 | -3.40--2.49 | -0.987 | -1.000--0.955 | <0.001 |
| FKGL | ChatGPT vs Gemini | 65 | 1.75 | 0.70-2.86 | 1.10 | 0.12-1.97 | 0.401 | 0.136-0.629 | 0.006 |
| FKGL | ChatGPT vs Copilot | 65 | -2.93 | -4.48--1.59 | -2.06 | -3.68--0.42 | -0.535 | -0.735--0.288 | <0.001 |
| FKGL | ChatGPT vs DeepSeek | 65 | 2.75 | 1.84-3.70 | 1.68 | 1.16-3.70 | 0.707 | 0.500-0.865 | <0.001 |
| FKGL | ChatGPT vs Doubao | 65 | -5.87 | -8.29--3.75 | -2.96 | -5.07--1.28 | -0.651 | -0.825--0.442 | <0.001 |
| FKGL | Gemini vs Copilot | 65 | -4.68 | -5.92--3.60 | -4.44 | -5.35--2.63 | -0.897 | -0.988--0.760 | <0.001 |
| FKGL | Gemini vs DeepSeek | 65 | 1.00 | 0.06-1.86 | 1.09 | 0.32-2.03 | 0.442 | 0.185-0.685 | 0.002 |
| FKGL | Gemini vs Doubao | 65 | -7.62 | -10.20--5.29 | -3.72 | -5.67--2.25 | -0.842 | -0.952--0.696 | <0.001 |
| FKGL | Copilot vs DeepSeek | 65 | 5.68 | 4.38-7.06 | 4.56 | 3.78-5.83 | 0.923 | 0.824-0.997 | <0.001 |
| FKGL | DeepSeek vs Doubao | 65 | -8.62 | -10.94--6.45 | -5.05 | -6.64--3.36 | -0.978 | -1.000--0.922 | <0.001 |
| GFI | ChatGPT vs Gemini | 65 | 1.71 | 0.66-2.86 | 0.71 | -0.17-2.61 | 0.332 | 0.064-0.572 | 0.023 |
| GFI | ChatGPT vs Copilot | 65 | -3.24 | -4.77--1.80 | -1.91 | -4.83--0.92 | -0.586 | -0.785--0.358 | <0.001 |
| GFI | ChatGPT vs DeepSeek | 65 | 2.78 | 1.84-3.78 | 1.79 | 0.94-3.67 | 0.704 | 0.501-0.865 | <0.001 |
| GFI | ChatGPT vs Doubao | 65 | -6.36 | -8.89--4.06 | -3.27 | -5.97--1.50 | -0.684 | -0.841--0.488 | <0.001 |
| GFI | Gemini vs Copilot | 65 | -4.95 | -6.17--3.80 | -4.35 | -5.68--3.39 | -0.905 | -0.991--0.780 | <0.001 |
| GFI | Gemini vs DeepSeek | 65 | 1.08 | 0.16-1.95 | 1.16 | 0.36-1.88 | 0.454 | 0.197-0.691 | 0.002 |
| GFI | Gemini vs Doubao | 65 | -8.07 | -10.75--5.71 | -4.20 | -5.95--3.02 | -0.857 | -0.962--0.717 | <0.001 |
| GFI | Copilot vs DeepSeek | 65 | 6.02 | 4.70-7.47 | 4.98 | 4.41-5.99 | 0.923 | 0.822-0.992 | <0.001 |
| GFI | DeepSeek vs Doubao | 65 | -9.14 | -11.73--6.94 | -5.36 | -6.99--3.84 | -0.976 | -1.000--0.921 | <0.001 |
| SMOG | ChatGPT vs Gemini | 65 | 0.97 | 0.28-1.68 | 0.47 | -0.04-1.54 | 0.319 | 0.041-0.571 | 0.028 |
| SMOG | ChatGPT vs Copilot | 65 | -2.37 | -3.26--1.52 | -1.80 | -3.43--0.64 | -0.674 | -0.838--0.465 | <0.001 |
| SMOG | ChatGPT vs DeepSeek | 65 | 1.77 | 1.12-2.43 | 1.35 | 0.57-2.62 | 0.686 | 0.468-0.856 | <0.001 |
| SMOG | ChatGPT vs Doubao | 65 | -3.72 | -5.00--2.50 | -1.87 | -4.12--0.95 | -0.710 | -0.866--0.514 | <0.001 |
| SMOG | Gemini vs Copilot | 65 | -3.34 | -4.05--2.62 | -2.89 | -3.98--2.41 | -0.919 | -0.992--0.814 | <0.001 |
| SMOG | Gemini vs DeepSeek | 65 | 0.80 | 0.24-1.34 | 0.79 | 0.22-1.42 | 0.456 | 0.198-0.677 | 0.002 |
| SMOG | Gemini vs Doubao | 65 | -4.69 | -6.07--3.45 | -2.77 | -3.91--2.00 | -0.869 | -0.966--0.732 | <0.001 |
| SMOG | Copilot vs DeepSeek | 65 | 4.14 | 3.34-4.98 | 3.56 | 3.04-4.36 | 0.947 | 0.866-1.000 | <0.001 |
| SMOG | DeepSeek vs Doubao | 65 | -5.50 | -6.88--4.30 | -3.74 | -4.73--2.75 | -0.982 | -1.000--0.941 | <0.001 |
| FRES | ChatGPT vs Gemini | 65 | -6.57 | -9.95--3.39 | -5.87 | -9.16--0.67 | -0.502 | -0.706--0.259 | <0.001 |
| FRES | ChatGPT vs Copilot | 65 | 18.17 | 14.18-22.34 | 15.80 | 10.65-22.44 | 0.922 | 0.817-0.989 | <0.001 |
| FRES | ChatGPT vs DeepSeek | 65 | -8.46 | -11.46--5.32 | -7.05 | -11.98--3.50 | -0.648 | -0.826--0.430 | <0.001 |
| FRES | ChatGPT vs Doubao | 65 | 20.99 | 14.96-27.77 | 14.75 | 9.40-21.67 | 0.814 | 0.667-0.925 | <0.001 |
| FRES | Gemini vs Copilot | 65 | 24.74 | 21.11-28.45 | 25.19 | 18.89-29.63 | 0.984 | 0.949-1.000 | <0.001 |
| FRES | Gemini vs Doubao | 65 | 27.56 | 21.04-34.32 | 19.31 | 13.88-24.85 | 0.921 | 0.825-0.984 | <0.001 |
| FRES | Copilot vs DeepSeek | 65 | -26.64 | -30.49--22.92 | -25.37 | -29.06--22.24 | -0.998 | -1.000--0.989 | <0.001 |
| FRES | DeepSeek vs Doubao | 65 | 29.45 | 23.25-36.13 | 21.41 | 16.38-26.00 | 0.999 | 0.994-1.000 | <0.001 |

Note: Pairwise comparisons were performed using paired Wilcoxon signed-rank tests with Benjamini-Hochberg correction. Paired mean/median differences and rank-biserial correlations are reported with bootstrap 95% CIs. Only significant comparisons are shown.

## Table S4. Significant pairwise comparisons of safety

**Note**

No significant pairwise safety difference after Benjamini-Hochberg correction.

Note: Pairwise comparisons were performed using McNemar tests with Benjamini-Hochberg correction. Safe-rate differences and their bootstrap 95% CIs are reported. Only significant comparisons are shown.

## Table S5. All pairwise comparisons for non-binary outcomes

| **Metric** | **Comparison** | **N pairs** | **Mean difference** | **Mean difference 95% CI** | **Median difference** | **Median difference 95% CI** | **Rank-biserial correlation** | **Rank-biserial 95% CI** | **Adjusted P** | **Note** |
| --- | --- | --- | --- | --- | --- | --- | --- | --- | --- | --- |
| Accuracy | ChatGPT vs Gemini | 65 | 0.27 | 0.14-0.40 | 0.00 | 0.00-0.20 | 0.684 | 0.396-0.907 | 0.003 | Paired Wilcoxon signed-rank test |
| Accuracy | ChatGPT vs Copilot | 65 | 0.08 | -0.02-0.19 | 0.00 | 0.00-0.00 | 0.254 | -0.169-0.607 | 0.303 | Paired Wilcoxon signed-rank test |
| Accuracy | ChatGPT vs DeepSeek | 65 | 0.21 | 0.05-0.36 | 0.00 | 0.00-0.20 | 0.459 | 0.114-0.773 | 0.068 | Paired Wilcoxon signed-rank test |
| Accuracy | ChatGPT vs Doubao | 65 | 0.15 | 0.01-0.28 | 0.00 | 0.00-0.00 | 0.396 | 0.000-0.729 | 0.120 | Paired Wilcoxon signed-rank test |
| Accuracy | Gemini vs Copilot | 65 | -0.19 | -0.33--0.05 | 0.00 | 0.00-0.00 | -0.444 | -0.747--0.083 | 0.068 | Paired Wilcoxon signed-rank test |
| Accuracy | Gemini vs DeepSeek | 65 | -0.06 | -0.20-0.06 | 0.00 | 0.00-0.00 | -0.166 | -0.548-0.237 | 0.405 | Paired Wilcoxon signed-rank test |
| Accuracy | Gemini vs Doubao | 65 | -0.12 | -0.25-0.01 | 0.00 | 0.00-0.00 | -0.353 | -0.693-0.030 | 0.129 | Paired Wilcoxon signed-rank test |
| Accuracy | Copilot vs DeepSeek | 65 | 0.12 | -0.03-0.28 | 0.00 | 0.00-0.00 | 0.340 | -0.057-0.704 | 0.129 | Paired Wilcoxon signed-rank test |
| Accuracy | Copilot vs Doubao | 65 | 0.06 | -0.10-0.22 | 0.00 | 0.00-0.00 | 0.159 | -0.215-0.513 | 0.405 | Paired Wilcoxon signed-rank test |
| Accuracy | DeepSeek vs Doubao | 65 | -0.06 | -0.19-0.08 | 0.00 | 0.00-0.00 | -0.178 | -0.540-0.202 | 0.405 | Paired Wilcoxon signed-rank test |
| Empathy | ChatGPT vs Gemini | 65 | -0.45 | -0.59--0.30 | -0.20 | -0.80-0.00 | -0.791 | -0.942--0.564 | <0.001 | Paired Wilcoxon signed-rank test |
| Empathy | ChatGPT vs Copilot | 65 | 0.35 | 0.21-0.50 | 0.20 | 0.00-0.20 | 0.644 | 0.398-0.834 | <0.001 | Paired Wilcoxon signed-rank test |
| Empathy | ChatGPT vs DeepSeek | 65 | 0.01 | -0.14-0.15 | 0.00 | 0.00-0.00 | 0.031 | -0.296-0.394 | 0.871 | Paired Wilcoxon signed-rank test |
| Empathy | ChatGPT vs Doubao | 65 | 0.23 | 0.10-0.36 | 0.00 | 0.00-0.20 | 0.546 | 0.236-0.801 | 0.003 | Paired Wilcoxon signed-rank test |
| Empathy | Gemini vs Copilot | 65 | 0.80 | 0.64-0.96 | 1.00 | 0.80-1.00 | 0.967 | 0.909-0.995 | <0.001 | Paired Wilcoxon signed-rank test |
| Empathy | Gemini vs DeepSeek | 65 | 0.46 | 0.30-0.62 | 0.60 | 0.00-1.00 | 0.757 | 0.537-0.910 | <0.001 | Paired Wilcoxon signed-rank test |
| Empathy | Gemini vs Doubao | 65 | 0.68 | 0.53-0.83 | 0.80 | 0.40-1.00 | 0.980 | 0.930-1.000 | <0.001 | Paired Wilcoxon signed-rank test |
| Empathy | Copilot vs DeepSeek | 65 | -0.34 | -0.47--0.21 | -0.20 | -0.40-0.00 | -0.698 | -0.887--0.463 | <0.001 | Paired Wilcoxon signed-rank test |
| Empathy | Copilot vs Doubao | 65 | -0.12 | -0.24-0.01 | 0.00 | 0.00-0.00 | -0.356 | -0.709-0.013 | 0.073 | Paired Wilcoxon signed-rank test |
| Empathy | DeepSeek vs Doubao | 65 | 0.22 | 0.09-0.38 | 0.00 | 0.00-0.20 | 0.483 | 0.165-0.736 | 0.008 | Paired Wilcoxon signed-rank test |
| DISCERN | ChatGPT vs Gemini | 65 | 2.87 | 1.95-3.79 | 2.40 | 1.40-3.60 | 0.723 | 0.528-0.870 | <0.001 | Paired Wilcoxon signed-rank test |
| DISCERN | ChatGPT vs Copilot | 65 | 1.19 | 0.36-2.10 | 0.80 | 0.00-1.80 | 0.347 | 0.060-0.597 | 0.027 | Paired Wilcoxon signed-rank test |
| DISCERN | ChatGPT vs DeepSeek | 65 | 2.42 | 1.50-3.35 | 2.00 | 0.80-3.20 | 0.662 | 0.464-0.835 | <0.001 | Paired Wilcoxon signed-rank test |
| DISCERN | ChatGPT vs Doubao | 65 | 2.10 | 1.30-2.96 | 1.80 | 1.00-3.00 | 0.643 | 0.428-0.828 | <0.001 | Paired Wilcoxon signed-rank test |
| DISCERN | Gemini vs Copilot | 65 | -1.68 | -2.47--0.89 | -1.60 | -2.40--1.00 | -0.570 | -0.786--0.319 | <0.001 | Paired Wilcoxon signed-rank test |
| DISCERN | Gemini vs DeepSeek | 65 | -0.45 | -1.23-0.27 | 0.20 | -0.60-0.60 | -0.084 | -0.362-0.213 | 0.561 | Paired Wilcoxon signed-rank test |
| DISCERN | Gemini vs Doubao | 65 | -0.77 | -1.50--0.08 | -0.80 | -1.80-0.20 | -0.304 | -0.562--0.039 | 0.043 | Paired Wilcoxon signed-rank test |
| DISCERN | Copilot vs DeepSeek | 65 | 1.23 | 0.34-2.12 | 1.60 | 1.00-2.20 | 0.443 | 0.171-0.689 | 0.005 | Paired Wilcoxon signed-rank test |
| DISCERN | Copilot vs Doubao | 65 | 0.91 | -0.04-1.82 | 1.00 | 0.20-1.60 | 0.344 | 0.074-0.609 | 0.027 | Paired Wilcoxon signed-rank test |
| DISCERN | DeepSeek vs Doubao | 65 | -0.32 | -1.00-0.36 | -0.20 | -1.00-0.60 | -0.101 | -0.374-0.182 | 0.536 | Paired Wilcoxon signed-rank test |
| EQIP | ChatGPT vs Gemini | 65 | 3.54 | 2.12-5.03 | 4.00 | 1.00-5.00 | 0.632 | 0.408-0.808 | <0.001 | Paired Wilcoxon signed-rank test |
| EQIP | ChatGPT vs Copilot | 65 | 1.74 | 0.42-3.22 | 2.00 | 0.00-3.00 | 0.330 | 0.051-0.589 | 0.052 | Paired Wilcoxon signed-rank test |
| EQIP | ChatGPT vs DeepSeek | 65 | 2.28 | 0.92-3.80 | 2.00 | 0.00-3.00 | 0.473 | 0.195-0.709 | 0.010 | Paired Wilcoxon signed-rank test |
| EQIP | ChatGPT vs Doubao | 65 | 2.31 | 0.91-3.72 | 2.00 | 1.00-4.00 | 0.453 | 0.172-0.695 | 0.010 | Paired Wilcoxon signed-rank test |
| EQIP | Gemini vs Copilot | 65 | -1.80 | -3.00--0.65 | -2.00 | -4.00--1.00 | -0.382 | -0.634--0.118 | 0.022 | Paired Wilcoxon signed-rank test |
| EQIP | Gemini vs DeepSeek | 65 | -1.26 | -2.40--0.14 | -1.00 | -2.00-0.00 | -0.297 | -0.556--0.010 | 0.089 | Paired Wilcoxon signed-rank test |
| EQIP | Gemini vs Doubao | 65 | -1.23 | -2.43--0.05 | -1.00 | -2.00-1.00 | -0.271 | -0.529-0.024 | 0.097 | Paired Wilcoxon signed-rank test |
| EQIP | Copilot vs DeepSeek | 65 | 0.54 | -0.77-1.78 | 1.00 | -1.00-3.00 | 0.196 | -0.099-0.480 | 0.238 | Paired Wilcoxon signed-rank test |
| EQIP | Copilot vs Doubao | 65 | 0.57 | -0.80-1.86 | 0.00 | -1.00-2.00 | 0.143 | -0.152-0.426 | 0.386 | Paired Wilcoxon signed-rank test |
| EQIP | DeepSeek vs Doubao | 65 | 0.03 | -1.11-1.15 | 0.00 | -1.00-2.00 | 0.047 | -0.248-0.347 | 0.759 | Paired Wilcoxon signed-rank test |
| JAMA | ChatGPT vs Gemini | 65 | -0.31 | -0.46--0.18 | 0.00 | -0.20-0.00 | -0.715 | -0.905--0.449 | <0.001 | Paired Wilcoxon signed-rank test |
| JAMA | ChatGPT vs Copilot | 65 | -0.89 | -0.98--0.78 | -1.00 | -1.00--1.00 | -0.998 | -1.000--0.989 | <0.001 | Paired Wilcoxon signed-rank test |
| JAMA | ChatGPT vs DeepSeek | 65 | -0.30 | -0.44--0.18 | 0.00 | -0.20-0.00 | -0.782 | -0.940--0.523 | <0.001 | Paired Wilcoxon signed-rank test |
| JAMA | ChatGPT vs Doubao | 65 | -0.31 | -0.46--0.17 | 0.00 | -0.20-0.00 | -0.668 | -0.867--0.392 | <0.001 | Paired Wilcoxon signed-rank test |
| JAMA | Gemini vs Copilot | 65 | -0.58 | -0.71--0.45 | -0.80 | -1.00--0.20 | -0.934 | -1.000--0.805 | <0.001 | Paired Wilcoxon signed-rank test |
| JAMA | Gemini vs DeepSeek | 65 | 0.01 | -0.12-0.15 | 0.00 | 0.00-0.00 | 0.022 | -0.333-0.356 | 0.906 | Paired Wilcoxon signed-rank test |
| JAMA | Gemini vs Doubao | 65 | -0.00 | -0.12-0.13 | 0.00 | 0.00-0.00 | -0.067 | -0.457-0.319 | 0.819 | Paired Wilcoxon signed-rank test |
| JAMA | Copilot vs DeepSeek | 65 | 0.59 | 0.43-0.74 | 1.00 | 0.80-1.00 | 0.792 | 0.594-0.946 | <0.001 | Paired Wilcoxon signed-rank test |
| JAMA | Copilot vs Doubao | 65 | 0.58 | 0.44-0.70 | 0.80 | 0.60-1.00 | 0.886 | 0.729-0.998 | <0.001 | Paired Wilcoxon signed-rank test |
| JAMA | DeepSeek vs Doubao | 65 | -0.01 | -0.14-0.13 | 0.00 | 0.00-0.00 | -0.114 | -0.492-0.249 | 0.698 | Paired Wilcoxon signed-rank test |
| GQS | ChatGPT vs Gemini | 65 | 0.31 | 0.16-0.46 | 0.00 | 0.00-0.20 | 0.637 | 0.362-0.862 | 0.004 | Paired Wilcoxon signed-rank test |
| GQS | ChatGPT vs Copilot | 65 | -0.00 | -0.13-0.14 | 0.00 | 0.00-0.00 | 0.002 | -0.434-0.427 | 1.000 | Paired Wilcoxon signed-rank test |
| GQS | ChatGPT vs DeepSeek | 65 | 0.17 | 0.04-0.30 | 0.00 | 0.00-0.00 | 0.486 | 0.138-0.788 | 0.036 | Paired Wilcoxon signed-rank test |
| GQS | ChatGPT vs Doubao | 65 | 0.12 | -0.02-0.26 | 0.00 | 0.00-0.00 | 0.324 | -0.057-0.686 | 0.135 | Paired Wilcoxon signed-rank test |
| GQS | Gemini vs Copilot | 65 | -0.31 | -0.48--0.14 | 0.00 | -0.20-0.00 | -0.584 | -0.841--0.268 | 0.006 | Paired Wilcoxon signed-rank test |
| GQS | Gemini vs DeepSeek | 65 | -0.14 | -0.28-0.00 | 0.00 | 0.00-0.00 | -0.323 | -0.667-0.061 | 0.135 | Paired Wilcoxon signed-rank test |
| GQS | Gemini vs Doubao | 65 | -0.18 | -0.33--0.05 | 0.00 | 0.00-0.00 | -0.447 | -0.749--0.088 | 0.046 | Paired Wilcoxon signed-rank test |
| GQS | Copilot vs DeepSeek | 65 | 0.17 | 0.02-0.32 | 0.00 | 0.00-0.00 | 0.466 | 0.091-0.797 | 0.046 | Paired Wilcoxon signed-rank test |
| GQS | Copilot vs Doubao | 65 | 0.12 | -0.02-0.27 | 0.00 | 0.00-0.00 | 0.342 | -0.053-0.669 | 0.135 | Paired Wilcoxon signed-rank test |
| GQS | DeepSeek vs Doubao | 65 | -0.05 | -0.18-0.09 | 0.00 | 0.00-0.00 | -0.121 | -0.497-0.255 | 0.593 | Paired Wilcoxon signed-rank test |
| ARI | ChatGPT vs Gemini | 65 | 2.32 | 1.04-3.71 | 1.30 | 0.25-2.54 | 0.406 | 0.147-0.637 | 0.005 | Paired Wilcoxon signed-rank test |
| ARI | ChatGPT vs Copilot | 65 | -3.24 | -5.23--1.37 | -2.50 | -4.37-0.17 | -0.439 | -0.657--0.179 | 0.003 | Paired Wilcoxon signed-rank test |
| ARI | ChatGPT vs DeepSeek | 65 | 3.71 | 2.58-4.91 | 2.63 | 1.37-4.87 | 0.766 | 0.579-0.905 | <0.001 | Paired Wilcoxon signed-rank test |
| ARI | ChatGPT vs Doubao | 65 | -7.20 | -10.32--4.40 | -4.14 | -6.34--1.41 | -0.621 | -0.799--0.406 | <0.001 | Paired Wilcoxon signed-rank test |
| ARI | Gemini vs Copilot | 65 | -5.56 | -7.19--4.02 | -4.69 | -6.70--2.69 | -0.855 | -0.967--0.703 | <0.001 | Paired Wilcoxon signed-rank test |
| ARI | Gemini vs DeepSeek | 65 | 1.39 | 0.20-2.46 | 1.49 | 0.34-2.34 | 0.496 | 0.243-0.711 | <0.001 | Paired Wilcoxon signed-rank test |
| ARI | Gemini vs Doubao | 65 | -9.52 | -12.94--6.44 | -4.45 | -7.03--2.92 | -0.842 | -0.948--0.698 | <0.001 | Paired Wilcoxon signed-rank test |
| ARI | Copilot vs DeepSeek | 65 | 6.94 | 5.23-8.75 | 5.14 | 4.40-7.66 | 0.888 | 0.767-0.980 | <0.001 | Paired Wilcoxon signed-rank test |
| ARI | Copilot vs Doubao | 65 | -3.96 | -7.46--0.54 | -0.94 | -2.69-1.23 | -0.218 | -0.483-0.060 | 0.128 | Paired Wilcoxon signed-rank test |
| ARI | DeepSeek vs Doubao | 65 | -10.91 | -14.19--8.07 | -6.13 | -8.60--4.50 | -0.978 | -1.000--0.919 | <0.001 | Paired Wilcoxon signed-rank test |
| CL | ChatGPT vs Gemini | 65 | 0.88 | 0.51-1.24 | 0.99 | 0.59-1.52 | 0.594 | 0.375-0.791 | <0.001 | Paired Wilcoxon signed-rank test |
| CL | ChatGPT vs Copilot | 65 | -3.14 | -3.63--2.66 | -2.91 | -3.80--2.34 | -0.999 | -1.000--0.994 | <0.001 | Paired Wilcoxon signed-rank test |
| CL | ChatGPT vs DeepSeek | 65 | 0.99 | 0.60-1.36 | 1.10 | 0.62-1.52 | 0.661 | 0.437-0.859 | <0.001 | Paired Wilcoxon signed-rank test |
| CL | ChatGPT vs Doubao | 65 | -1.84 | -2.33--1.38 | -2.00 | -2.41--1.26 | -0.824 | -0.932--0.678 | <0.001 | Paired Wilcoxon signed-rank test |
| CL | Gemini vs Copilot | 65 | -4.02 | -4.52--3.51 | -3.94 | -4.59--3.40 | -1.000 | -1.000--1.000 | <0.001 | Paired Wilcoxon signed-rank test |
| CL | Gemini vs DeepSeek | 65 | 0.11 | -0.29-0.49 | 0.07 | -0.41-0.63 | 0.086 | -0.195-0.367 | 0.548 | Paired Wilcoxon signed-rank test |
| CL | Gemini vs Doubao | 65 | -2.71 | -3.21--2.21 | -2.74 | -3.19--2.27 | -0.956 | -0.993--0.892 | <0.001 | Paired Wilcoxon signed-rank test |
| CL | Copilot vs DeepSeek | 65 | 4.13 | 3.67-4.61 | 4.26 | 3.82-4.95 | 0.999 | 0.994-1.000 | <0.001 | Paired Wilcoxon signed-rank test |
| CL | Copilot vs Doubao | 65 | 1.30 | 0.80-1.79 | 1.27 | 0.81-2.00 | 0.647 | 0.442-0.840 | <0.001 | Paired Wilcoxon signed-rank test |
| CL | DeepSeek vs Doubao | 65 | -2.82 | -3.19--2.43 | -2.93 | -3.40--2.49 | -0.987 | -1.000--0.955 | <0.001 | Paired Wilcoxon signed-rank test |
| FKGL | ChatGPT vs Gemini | 65 | 1.75 | 0.70-2.86 | 1.10 | 0.12-1.97 | 0.401 | 0.136-0.629 | 0.006 | Paired Wilcoxon signed-rank test |
| FKGL | ChatGPT vs Copilot | 65 | -2.93 | -4.48--1.59 | -2.06 | -3.68--0.42 | -0.535 | -0.735--0.288 | <0.001 | Paired Wilcoxon signed-rank test |
| FKGL | ChatGPT vs DeepSeek | 65 | 2.75 | 1.84-3.70 | 1.68 | 1.16-3.70 | 0.707 | 0.500-0.865 | <0.001 | Paired Wilcoxon signed-rank test |
| FKGL | ChatGPT vs Doubao | 65 | -5.87 | -8.29--3.75 | -2.96 | -5.07--1.28 | -0.651 | -0.825--0.442 | <0.001 | Paired Wilcoxon signed-rank test |
| FKGL | Gemini vs Copilot | 65 | -4.68 | -5.92--3.60 | -4.44 | -5.35--2.63 | -0.897 | -0.988--0.760 | <0.001 | Paired Wilcoxon signed-rank test |
| FKGL | Gemini vs DeepSeek | 65 | 1.00 | 0.06-1.86 | 1.09 | 0.32-2.03 | 0.442 | 0.185-0.685 | 0.002 | Paired Wilcoxon signed-rank test |
| FKGL | Gemini vs Doubao | 65 | -7.62 | -10.20--5.29 | -3.72 | -5.67--2.25 | -0.842 | -0.952--0.696 | <0.001 | Paired Wilcoxon signed-rank test |
| FKGL | Copilot vs DeepSeek | 65 | 5.68 | 4.38-7.06 | 4.56 | 3.78-5.83 | 0.923 | 0.824-0.997 | <0.001 | Paired Wilcoxon signed-rank test |
| FKGL | Copilot vs Doubao | 65 | -2.95 | -5.79--0.43 | -0.02 | -1.75-1.02 | -0.173 | -0.428-0.119 | 0.227 | Paired Wilcoxon signed-rank test |
| FKGL | DeepSeek vs Doubao | 65 | -8.62 | -10.94--6.45 | -5.05 | -6.64--3.36 | -0.978 | -1.000--0.922 | <0.001 | Paired Wilcoxon signed-rank test |
| GFI | ChatGPT vs Gemini | 65 | 1.71 | 0.66-2.86 | 0.71 | -0.17-2.61 | 0.332 | 0.064-0.572 | 0.023 | Paired Wilcoxon signed-rank test |
| GFI | ChatGPT vs Copilot | 65 | -3.24 | -4.77--1.80 | -1.91 | -4.83--0.92 | -0.586 | -0.785--0.358 | <0.001 | Paired Wilcoxon signed-rank test |
| GFI | ChatGPT vs DeepSeek | 65 | 2.78 | 1.84-3.78 | 1.79 | 0.94-3.67 | 0.704 | 0.501-0.865 | <0.001 | Paired Wilcoxon signed-rank test |
| GFI | ChatGPT vs Doubao | 65 | -6.36 | -8.89--4.06 | -3.27 | -5.97--1.50 | -0.684 | -0.841--0.488 | <0.001 | Paired Wilcoxon signed-rank test |
| GFI | Gemini vs Copilot | 65 | -4.95 | -6.17--3.80 | -4.35 | -5.68--3.39 | -0.905 | -0.991--0.780 | <0.001 | Paired Wilcoxon signed-rank test |
| GFI | Gemini vs DeepSeek | 65 | 1.08 | 0.16-1.95 | 1.16 | 0.36-1.88 | 0.454 | 0.197-0.691 | 0.002 | Paired Wilcoxon signed-rank test |
| GFI | Gemini vs Doubao | 65 | -8.07 | -10.75--5.71 | -4.20 | -5.95--3.02 | -0.857 | -0.962--0.717 | <0.001 | Paired Wilcoxon signed-rank test |
| GFI | Copilot vs DeepSeek | 65 | 6.02 | 4.70-7.47 | 4.98 | 4.41-5.99 | 0.923 | 0.822-0.992 | <0.001 | Paired Wilcoxon signed-rank test |
| GFI | Copilot vs Doubao | 65 | -3.12 | -5.90--0.51 | -0.52 | -1.78-0.84 | -0.201 | -0.468-0.073 | 0.160 | Paired Wilcoxon signed-rank test |
| GFI | DeepSeek vs Doubao | 65 | -9.14 | -11.73--6.94 | -5.36 | -6.99--3.84 | -0.976 | -1.000--0.921 | <0.001 | Paired Wilcoxon signed-rank test |
| SMOG | ChatGPT vs Gemini | 65 | 0.97 | 0.28-1.68 | 0.47 | -0.04-1.54 | 0.319 | 0.041-0.571 | 0.028 | Paired Wilcoxon signed-rank test |
| SMOG | ChatGPT vs Copilot | 65 | -2.37 | -3.26--1.52 | -1.80 | -3.43--0.64 | -0.674 | -0.838--0.465 | <0.001 | Paired Wilcoxon signed-rank test |
| SMOG | ChatGPT vs DeepSeek | 65 | 1.77 | 1.12-2.43 | 1.35 | 0.57-2.62 | 0.686 | 0.468-0.856 | <0.001 | Paired Wilcoxon signed-rank test |
| SMOG | ChatGPT vs Doubao | 65 | -3.72 | -5.00--2.50 | -1.87 | -4.12--0.95 | -0.710 | -0.866--0.514 | <0.001 | Paired Wilcoxon signed-rank test |
| SMOG | Gemini vs Copilot | 65 | -3.34 | -4.05--2.62 | -2.89 | -3.98--2.41 | -0.919 | -0.992--0.814 | <0.001 | Paired Wilcoxon signed-rank test |
| SMOG | Gemini vs DeepSeek | 65 | 0.80 | 0.24-1.34 | 0.79 | 0.22-1.42 | 0.456 | 0.198-0.677 | 0.002 | Paired Wilcoxon signed-rank test |
| SMOG | Gemini vs Doubao | 65 | -4.69 | -6.07--3.45 | -2.77 | -3.91--2.00 | -0.869 | -0.966--0.732 | <0.001 | Paired Wilcoxon signed-rank test |
| SMOG | Copilot vs DeepSeek | 65 | 4.14 | 3.34-4.98 | 3.56 | 3.04-4.36 | 0.947 | 0.866-1.000 | <0.001 | Paired Wilcoxon signed-rank test |
| SMOG | Copilot vs Doubao | 65 | -1.35 | -2.83-0.08 | -0.35 | -1.16-0.66 | -0.186 | -0.462-0.087 | 0.193 | Paired Wilcoxon signed-rank test |
| SMOG | DeepSeek vs Doubao | 65 | -5.50 | -6.88--4.30 | -3.74 | -4.73--2.75 | -0.982 | -1.000--0.941 | <0.001 | Paired Wilcoxon signed-rank test |
| FRES | ChatGPT vs Gemini | 65 | -6.57 | -9.95--3.39 | -5.87 | -9.16--0.67 | -0.502 | -0.706--0.259 | <0.001 | Paired Wilcoxon signed-rank test |
| FRES | ChatGPT vs Copilot | 65 | 18.17 | 14.18-22.34 | 15.80 | 10.65-22.44 | 0.922 | 0.817-0.989 | <0.001 | Paired Wilcoxon signed-rank test |
| FRES | ChatGPT vs DeepSeek | 65 | -8.46 | -11.46--5.32 | -7.05 | -11.98--3.50 | -0.648 | -0.826--0.430 | <0.001 | Paired Wilcoxon signed-rank test |
| FRES | ChatGPT vs Doubao | 65 | 20.99 | 14.96-27.77 | 14.75 | 9.40-21.67 | 0.814 | 0.667-0.925 | <0.001 | Paired Wilcoxon signed-rank test |
| FRES | Gemini vs Copilot | 65 | 24.74 | 21.11-28.45 | 25.19 | 18.89-29.63 | 0.984 | 0.949-1.000 | <0.001 | Paired Wilcoxon signed-rank test |
| FRES | Gemini vs DeepSeek | 65 | -1.89 | -4.95-1.17 | -1.09 | -5.57-0.78 | -0.206 | -0.470-0.081 | 0.165 | Paired Wilcoxon signed-rank test |
| FRES | Gemini vs Doubao | 65 | 27.56 | 21.04-34.32 | 19.31 | 13.88-24.85 | 0.921 | 0.825-0.984 | <0.001 | Paired Wilcoxon signed-rank test |
| FRES | Copilot vs DeepSeek | 65 | -26.64 | -30.49--22.92 | -25.37 | -29.06--22.24 | -0.998 | -1.000--0.989 | <0.001 | Paired Wilcoxon signed-rank test |
| FRES | Copilot vs Doubao | 65 | 2.82 | -4.35-10.12 | -3.50 | -6.39-2.55 | -0.054 | -0.342-0.228 | 0.705 | Paired Wilcoxon signed-rank test |
| FRES | DeepSeek vs Doubao | 65 | 29.45 | 23.25-36.13 | 21.41 | 16.38-26.00 | 0.999 | 0.994-1.000 | <0.001 | Paired Wilcoxon signed-rank test |

Note: Pairwise comparisons were performed using paired Wilcoxon signed-rank tests with Benjamini-Hochberg correction. Paired mean/median differences and rank-biserial correlations are reported with bootstrap 95% CIs.
